# Supplementary figures and images for: Type 1 diabetes contributes to combined pulmonary fibrosis and emphysema in male alpha 1 antitrypsin deficient mice
Source: PLoS One. 2023 Oct 11;18(10):e0291948. doi: 10.1371/journal.pone.0291948 (PMC10566687; doi:10.1371/journal.pone.0291948)

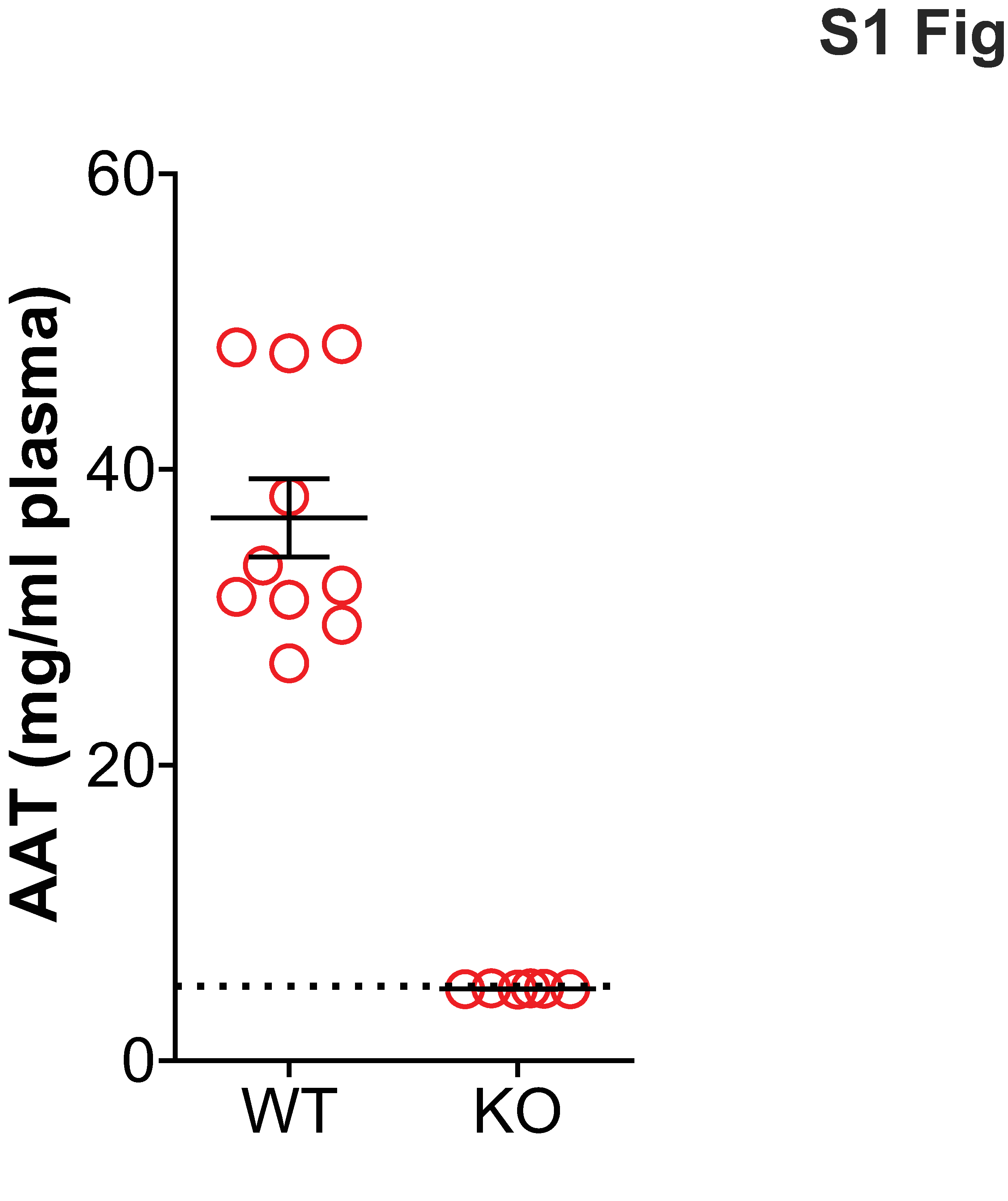

Supplement: S1 Fig — (A) Plasma concentration of AAT was measured in control and STZ mice using a commercially available ELISA. The dotted lines denote the limit of detection of the assay. The data are expressed as dot plots with the means ± S.E.M. (TIF) [file pone.0291948.s001.tif]

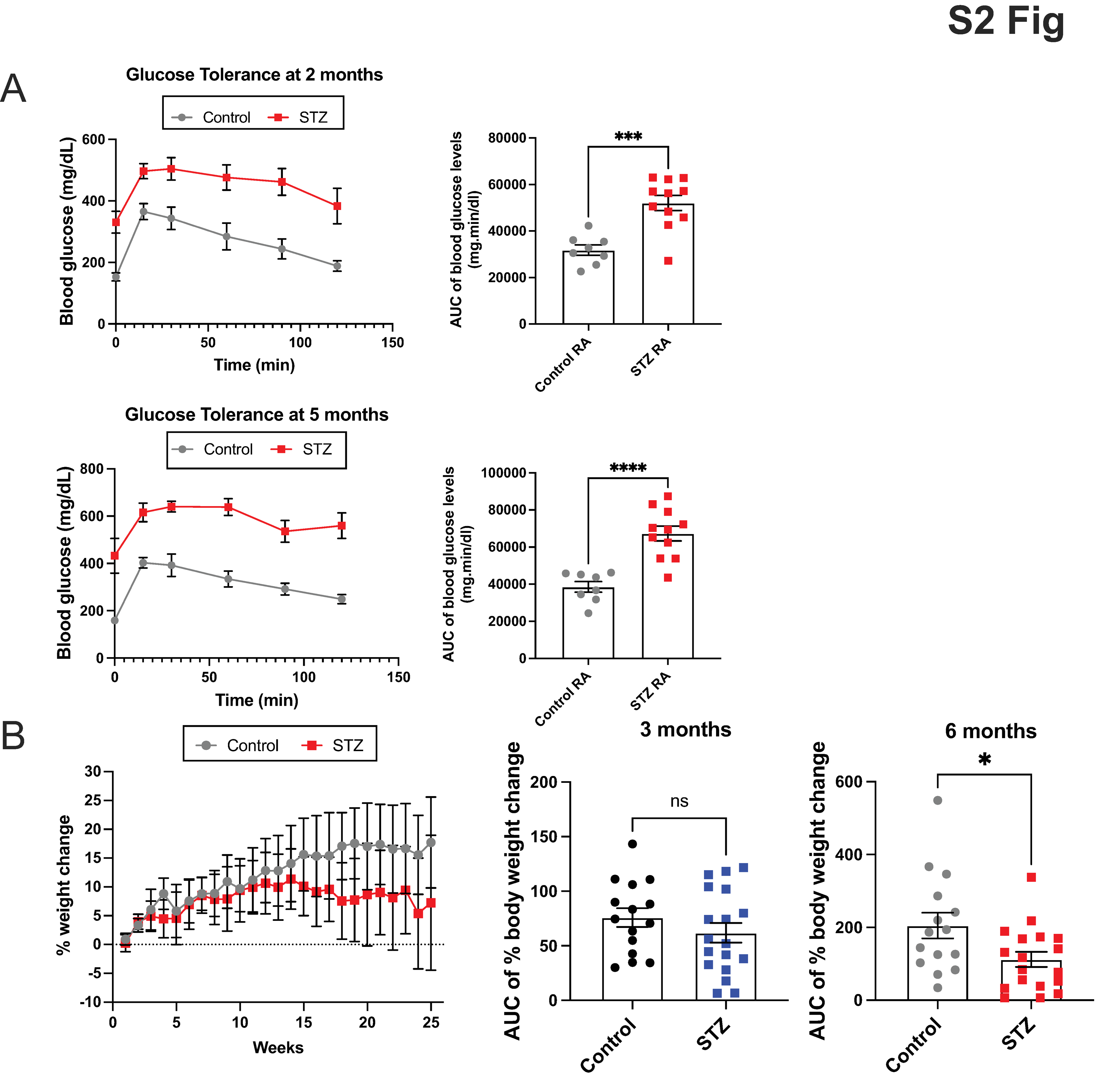

Supplement: S2 Fig — (A) Mice were challenged with glucose (2 g/kg, i.p.), and fasting blood glucose was measured at 0, 15, 30, 60, 90 and 120 min after the challenge. Glucose challenge was performed at 2 months and 5 months since STZ or citrate buffer injections. (B) Body weights of the mice were measured weekly and % body weight change was calculated. (A-B) The bar graphs show the area under the curve (AUC) of curves. Data were analyzed by unpaired t-test. N = 8–18. *p≤0.05, **p≤0.01. (TIF) [file pone.0291948.s002.tif]

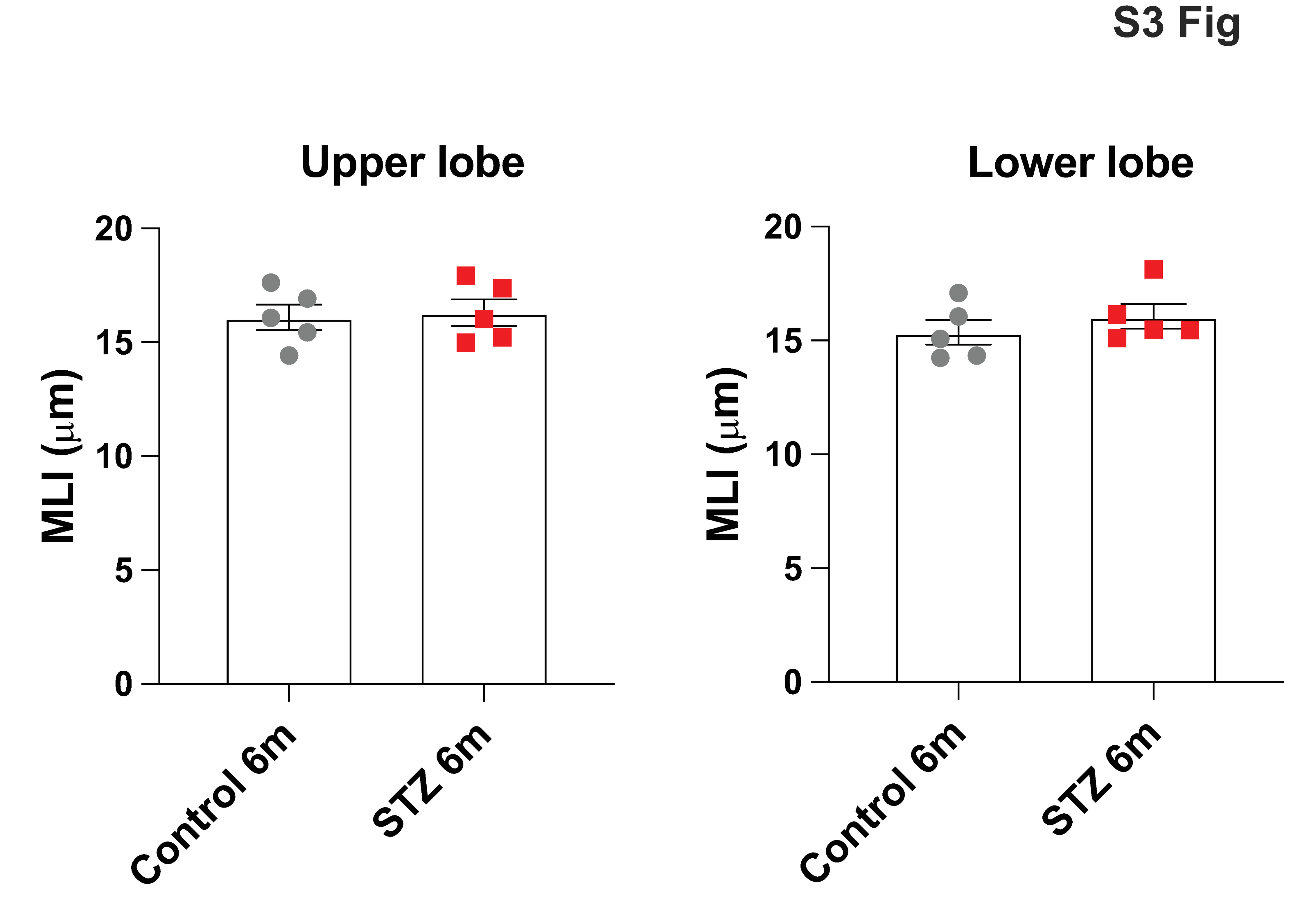

Supplement: S3 Fig — Mean free distance in the airspace was assessed by mean linear intercept measurements in the upper and lower lobes of the lung in control or STZ mice exposed to RA or CS for 6 months. Data were analyzed by two-way ANOVA using Tukey’s post hoc tests. N = 5/group. (TIF) [file pone.0291948.s003.tif]

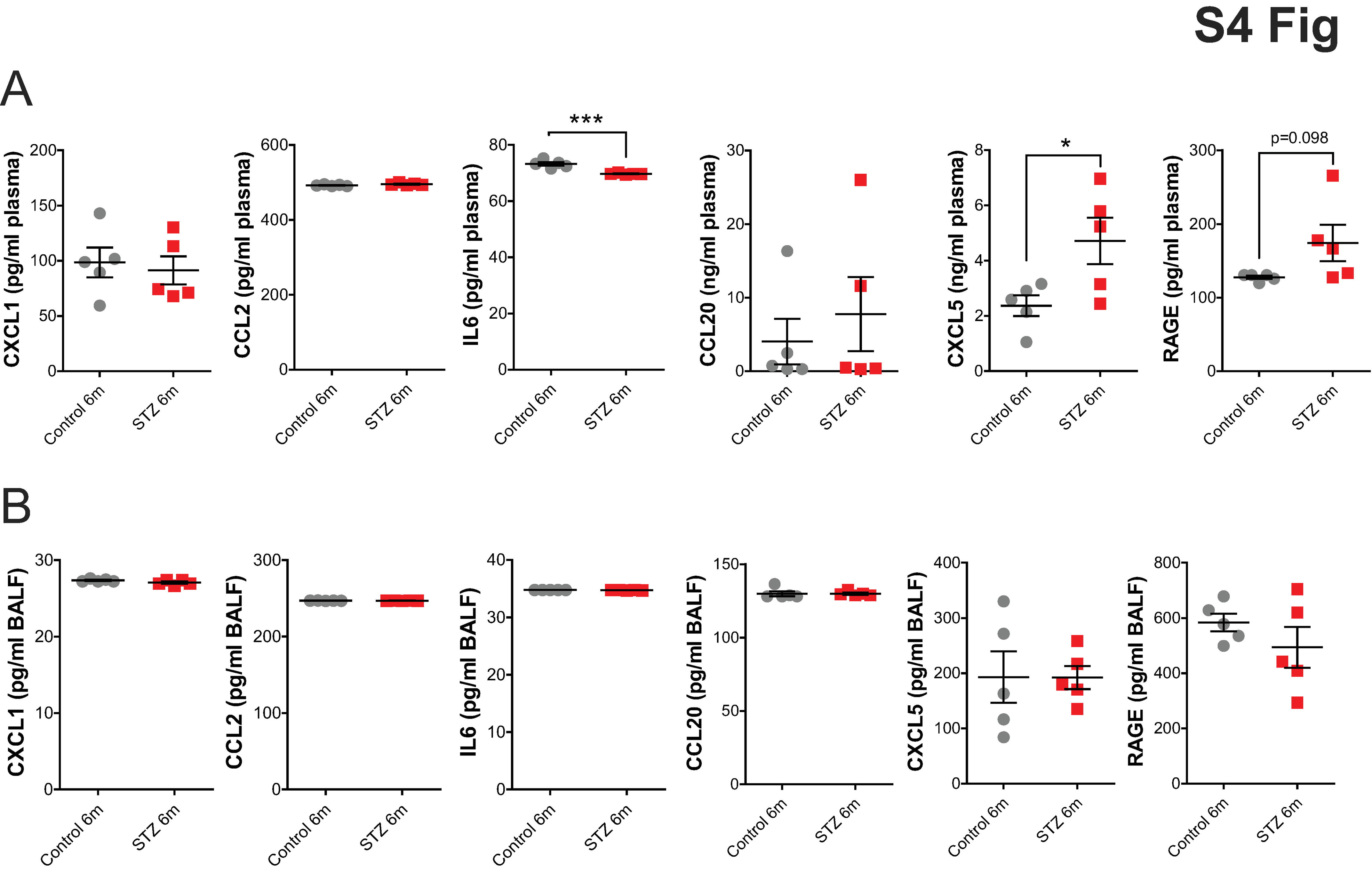

Supplement: S4 Fig — Protein concentrations of CXCL1, CCL2, IL-6, CCL20, CXCL5, and RAGE were measured by Luminex multiplex assay in the A) plasma and B) BAL samples from control and STZ mice. Data were analyzed by Student’s t-test. *p≤0.05; **p≤0.01; ***p≤0.001. (TIF) [file pone.0291948.s004.tif]
