# Supplementary material for: Type 1 diabetes contributes to combined pulmonary fibrosis and emphysema in male alpha 1 antitrypsin deficient mice
Source: PLoS One. 2023 Oct 11;18(10):e0291948. doi: 10.1371/journal.pone.0291948 (PMC10566687; doi:10.1371/journal.pone.0291948)
Supplement: S1 Table — (DOCX) [file pone.0291948.s005.docx]

**S1 Table. Primer details**

| **Gene Symbol** | **Species** | **Gene Name** | **Primer Pair** |
| --- | --- | --- | --- |
| *Hprt1* | Mouse | Hypoxanthine phosphoribosyltransferase 1 | FWD: 5’ AGCCTAAGATGAGCGCAAGT 3’  REV: 5’ GCCACAGGACTAGAACACCT 3’ |
| *Fn1* | Mouse | Fibronectin 1 | FWD: 5’ GGTCAGTCCTACAAGATT 3’  REV: 5’ ACACAGACACTCTAACAT 3’ |
| *Acta2* | Mouse | Actin alpha 2, smooth muscle | FWD: 5’ TGGCATCAATCACTTCAA 3’  REV: 5’ CCTATCTGGTCACCTGTA 3’ |
| *Ccn2* | Mouse | Cellular communication network factor 2 | FWD: 5’ ACACGAACTCATTAGACTAT 3’  REV: 5’ AGGTTGACAGACTACTTG 3’ |
| *HPRT1* | Human | Hypoxanthine phosphoribosyltransferase 1 | FWD: 5’ GACAGGACTGAACGTCTTGC 3’  REV: 5’ GCACACAGAGGGCTACAATG 3’ |
| *FN1* | Human | Fibronectin 1 | FWD: 5’ AATAATCAGAAGAGCGAG 3’  REV: 5’ GAATACCATTTCCAGTGT 3’ |
| *ACTA2* | Human | Actin alpha 2, smooth muscle | FWD: 5’ TGATTAAGGTGGAGGAGTAA 3’  REV: 5’ TGATGATGATGATGATGATGAT 3’ |
| *CCN2* | Human | Cellular communication network factor 2 | FWD: 5’ TTCAGTAGCACAAGTTAT 3’  REV: 5’ TTCAGTAGCACAAGTTAT 3’ |
